# Photodegradation of Eosin Y Using Silver-Doped Magnetic Nanoparticles

**DOI:** 10.1155/2015/797606

**Published:** 2015-11-04

**Authors:** Eman Alzahrani

**Affiliations:** Chemistry Department, Faculty of Science, Taif University, P.O. Box 888, Taif, Saudi Arabia

## Abstract

The purification of industrial wastewater from dyes is becoming increasingly important since they are toxic or carcinogenic to human beings. Nanomaterials have been receiving significant attention due to their unique physical and chemical properties compared with their larger-size counterparts. The aim of the present investigation was to fabricate magnetic nanoparticles (MNPs) using a coprecipitation method, followed by coating with silver (Ag) in order to enhance the photocatalytic activity of the MNPs by loading metal onto them. The fabricated magnetic nanoparticles coated with Ag were characterised using different instruments such as a scanning electron microscope (SEM), transmission electron microscopy (TEM), energy-dispersive X-ray (EDAX) spectroscopy, and X-ray diffraction (XRD) analysis. The average size of the magnetic nanoparticles had a mean diameter of about 48 nm, and the average particle size changed to 55 nm after doping. The fabricated Ag-doped magnetic nanoparticles were used for the degradation of eosin Y under UV-lamp irradiation. The experimental results revealed that the use of fabricated magnetic nanoparticles coated with Ag can be considered as reliable methods for the removal of eosin Y since the slope of evaluation of pseudo-first-order rate constant from the slope of the plot between ln⁡(*C*_*o*_/*C*) and the irradiation time was found to be linear. Ag-Fe_3_O_4_ nanoparticles would be considered an efficient photocatalyst to degrade textile dyes avoiding the tedious filtration step.

## 1. Introduction

In recent decades, environmental problems have attracted increasing attention all over the world. One type of environmental pollutants of concern is synthetic dyes, which are commonly used for colouring materials such as textiles, leather, paper, wool, printed matter, and cosmetics [1, 2]. Although the exact number of the produced dyes in the world is not known, it is estimated that there are more than 100,000 different available dyes. These dyes are difficult to decolourise because of their complex aromatic structure, which originates from coal-tar-based hydrocarbons such as benzene, naphthalene, anthracene, toluene, and xylene [3–8]. Commonly, these dyes are discharged into the environment in the form of coloured wastewater by many industries without any prior treatment; however, many of them are known to be toxic or carcinogenic to human beings [9–11]. Therefore, several researchers are working on methods for the removal of dyes from wastewater before their discharge into downstream bodies of water [12–16].

There are several methods that have been utilised for the removal of dyes from industrial wastewater, for example, adsorption, nanofiltration, ozonation, and electroflotation [17–19]. All of these processes have different capabilities for removing dyes. Among these methods, adsorption has been found to be the best technique for wastewater purification [20–22], due to the simplicity of the procedure, ease of operation, and its nonsensitivity to toxic substances. The purification of wastewater from harmful dyes can be performed using activated carbon, which has a high surface area [23–26]; however, it also has a high waste cost. Other materials that can be utilised are alum, ferric salt, or lime. Although the cost of these materials is cheap, they suffer from several severe drawbacks [27–30].

The field of nanotechnology is one of the most active areas of research in modern materials science. The application of nanoscale materials, which range from 1 to 100 nm, is a key emerging area of nanotechnology. There is an increased demand for nanoparticles because of their wide applicability in various areas, for example, electronics, catalysis, chemistry, energy, and medicine [31–34].

Magnetic nanoparticles (MNPs) are a type of nanoparticles that can be manipulated using a magnetic field. MNPs are attracting significant attention due to their large surface area, high magnetic properties, high thermal stability, high mechanical strength, and lack of toxicity [35–37]. Moreover, MNPs can be rapidly prepared, have high separation efficiency, are cost-effective, and provide for a simple operational process. They are utilised in applications such as magnetic drug targeting, magnetic resonance imaging for clinical diagnosis, recording material, and biomedical applications [38–41].

MNPs possessing magnetite (Fe_3_O_4_) or maghemite (*γ*-Fe_2_O_3_) core chemistry have been used to solve a range of environmental problems; for example, MNPs have been utilised for the purification of wastewater by removing heavy metals, alkalinity, and hardness, as well as natural organic compounds and salt. This is due to their simple fabrication, easy optimisation of their size and morphology, and fast magnetic separation under an external magnetic field [42–45].

Iron oxide nanoparticles can be fabricated using different methods, for example, coprecipitation, energy milling, reduction, and ultrasonic-assisted impregnation. The coprecipitation method is based on mixing ferrous and ferric ions in the ratio of 1 to 2 in an alkaline medium. The main advantage of this method is that it produces fine and stoichiometric particles of single and multicomponent metal oxides [46–50].

In order to improve the physicochemical properties of MNPs and to achieve different kinds of applications, modification of the surfaces of MNPs with functional groups is necessary [51, 52]. The doping of MNPs with metals can resolve the problem of the fast recombination of charge carriers, which happens within nanoseconds, by trapping and subsequently transferring the photoexcited electrons onto the surface of photocatalyst, resulting in decreased recombination of the electron-hole pairs [53, 54].

According to our literature survey, only very few studies have been carried out on the use of MNPs doping with metal for the photodegradation of harmful dyes. Therefore, the purpose of the present paper is to describe the fabrication of magnetic nanoparticles (MNPs) using the precipitation method, followed by doping of the MNPs with Ag. Characterisations of the fabricated Ag-doped magnetic nanoparticles were performed by studying the physical properties of the fabricated nanoparticles using scanning electron microscopy (SEM) analysis, transmission electron microscopy (TEM) analysis, energy-dispersive X-ray analysis (EDAX), and X-ray diffraction (XRD) analysis. The fabricated materials were used for the photodegradation of a model dye, namely, eosin Y (the characteristics of eosin Y are given in Table 1), in the presence of ultraviolet light (UV). Furthermore, the photocatalytic activity of the Ag-MNPs was compared with naked MNPs. Moreover, the degradation kinetics were followed to study the photocatalytic efficiency of the Ag-MNPs.

## 2. Experimental

### 2.1. Chemicals and Materials

Iron(II) sulfate heptahydrate (FeSO_4_·7H_2_O, 98%), Mwt. = 151.91 g mol^−1^, sodium nitrite (NaNO_3_, 99%), and eosin Y were purchased from Sigma-Aldrich (Nottingham, UK). Sodium hydroxide (NaOH) was purchased from Loba Chemie Pvt. Ltd. (Mumbai, India). Silver nitrate (AgNO_3_, 99.85%) and sodium carbonate (Na_2_CO_3_) were purchased from Acros Organics (Loughborough, UK). Cylindrical rod magnets (40 mm diameter × 40 mm thickness) for settlement of the magnetic nanoparticles were purchased from Magnet Expert Ltd. (Tuxford, UK). Distilled water was employed for preparing all the solutions and reagents.

### 2.2. Instrumentation

The magnetic stirrer and heater were purchased from Fisher Scientific Co. Ltd. (Shanghai, China). The oven was from Memmert (Nuremberg, Germany). X-ray diffraction (XRD) patterns were obtained using a Bruker diffractometer D8-ADVANCE with CuK_*α*1_ radiation (Coventry, UK). The transmission electron microscope (TEM) came from JEOL Ltd. (Welwyn Garden City, UK). The scanning electron microscope (SEM) with unit energy-dispersive X-ray analysis (EDAX) was obtained from JEOL JSM 6390 LA Analytical (Tokyo, Japan). The UV lamp (*λ* = 365 nm) was purchased from Spectronic Analytical Instruments (Leeds, UK). The UV-Vis spectrophotometer was from Thermo Scientific GENESYS 10S (Toronto, Canada).

### 2.3. Preparation of the Magnetic Iron Oxide Nanoparticles

Magnetic nanoparticles (MNPs) were prepared as described by Tan and Bakar [55], with some modification. MNPs were obtained by dissolving 3.3 g of FeSO_4_·7H_2_O and 2 g of NaNO_3_ in 50 mL of distilled water. Then, 20 mL of NaOH solution (2.5 M) was added to the mixture while it was heated up to 80°C. The reaction was allowed to proceed at 80°C under constant stirring to ensure the complete growth of the nanoparticle crystals. After 30 minutes, the resulting suspension was cooled down to room temperature and washed with distilled water repeatedly to remove unreacted chemicals. The Fe_3_O_4_ magnetic nanoparticles were separated using an external magnet and dried in an oven at 50°C overnight before the coating.

### 2.4. Doping of MNPs with Ag

The prepared magnetic nanoparticles were doped with Ag. This was performed by mixing 10 g of magnetic nanoparticles with 10 mL of silver nitrate solution (0.1 M). Then, 10 mL of sodium carbonate (1%) solution was added. The suspension was dried at 80°C for 24 h, and finally the formed powder was calcinated at 400°C for 6 h in a furnace. The formed Ag-doped MNPs were washed with distilled water. The product was dried at room temperature for 12 h in a vacuum desiccator.

### 2.5. Characterisation of the Prepared Materials

The morphology and mean size of the samples were determined by transmission electron microscopy (TEM). A drop of well-dispersed nanoparticle dispersion was placed onto the amorphous carbon-coated 200-mesh copper grid and allowed to dry at ambient temperature, and then the grid was scanned. The average size of about 100 nanoparticles of each sample was measured from the TEM images by using the ImageJ software. The morphology of the fabricated magnetic nanoparticles before and after doping with Ag was characterised by SEM analysis. The compositional analysis was performed using energy-dispersive X-ray analysis (EDAX). Phase identification and structural analysis of the magnetic nanoparticles were carried out using X-ray diffraction (XRD) with the CuK_*α*_ radiation in the 2-theta range from 20° to 70°.

### 2.6. Photocatalytic Degradation of Eosin Y

The photocatalytic activity of Ag-doped Fe_3_O_4_ nanoparticles was investigated by the decolourisation of eosin Y in aqueous solution. All the degradation reactions were performed according to the following procedure: 50 mg of photocatalyst was added to 100 mL of aqueous dye solution (4 × 10^−4^ M) placed in a cylindrical glass vessel. Before exposure to the UV lamp, the suspension was magnetically stirred at 600 rpm for 1 h in the dark in order to achieve the adsorption-desorption equilibrium between the photocatalyst and the dye. The reaction was performed with constant magnetic stirring during the reaction. A 2 mL aliquot was withdrawn from the mixture solution at 60 min intervals. The degradation of eosin Y was spectrometrically monitored with a UV-Vis spectrophotometer at a wavelength between 350 and 800 nm according to the dye. The photocatalyst was precipitated from the solution by applying an external magnet and then washed with distilled water and stored for further use. The efficiency of the photocatalytic degradation of the fabricated materials was calculated based on the degree of absorption in the absorption spectra of the dye solution with respect to the intensity corresponding to *λ*_max_ of eosin Y (515 nm) by using the equation given below [56–58]:

(1) $$\begin{array}{c} D\%=\frac{C_{o}-C}{C_{o}}\times 100=\frac{A_{o}-A}{A_{o}}\times 100, \end{array}$$

where *C*_*o*_ is the initial concentration of the dye and *C* is the concentration of the dye after irradiation in a selected time interval. Parameters *A*_*o*_ and *A* are the absorbance of eosin Y solutions in the 515 nm wavelength at the initial and at any time thereafter, respectively.

## 3. Results and Discussion 

### 3.1. Preparation of Fe_3_O_4_ and the Ag-Doped Fe_3_O_4_ Nanoparticles

In this study, magnetic nanoparticles (MNPs) were fabricated. The reason for choosing this type of nanoparticle was because it is easy to fabricate and it is easy to separate it from the solution by using an external magnet [38, 59, 60]. The chemical precipitation technique was used to prepare the magnetic nanoparticles. This technique is probably the most common and efficient method to obtain magnetic particles [40, 61, 62]. The fabrication of MNPs was performed by mixing iron(II) sulfate heptahydrate and sodium nitrite in purified water. Complete precipitation of Fe_3_O_4_ nanoparticles was achieved under alkaline condition, which was 20 mL of NaOH solution (2.5 M). When NaOH solution was added to the FeSO_4_, Fe(OH)_2_ was initially formed, which was then oxidised to the Fe_3_O_4_ nanoparticle [61, 63, 64]. Finally, the formed magnetic nanoparticles were separated from the reaction medium by a permanent magnet field and washed with distilled water to remove excess ions such as SO_4_^2−^ and NO_3_^−^.

**Figure 1:**
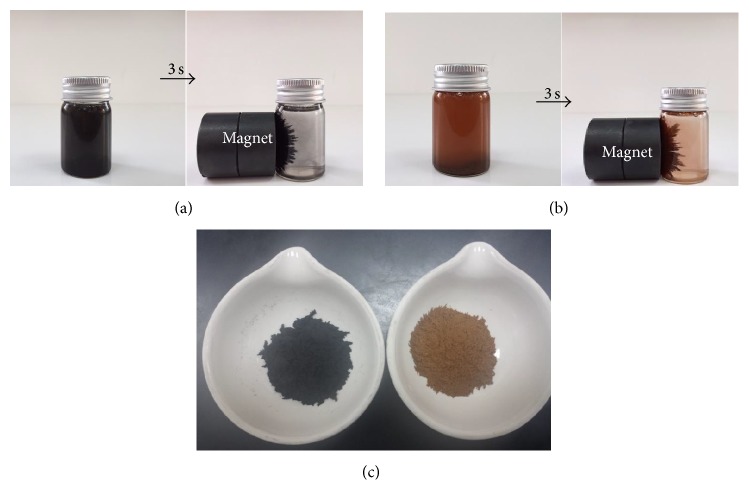
Photographs of aqueous solutions of F_3_O_4_ nanoparticles (a), Ag-Fe_3_O_4_ nanoparticles in a vial (b) before and after being separated from solution using an external magnet for 3 s, and (c) the dried fabricated materials.

The MNPs were doped with Ag in order to obtain a higher photocatalytic efficiency than the naked Fe_3_O_4_ nanoparticles. After calcination, the colour of the Fe_3_O_4_ nanoparticles turned from black to brownish.  Figure 1 shows photographs of aqueous solutions of the unsupported Fe_3_O_4_ nanoparticles (a) and Ag-doped Fe_3_O_4_ nanoparticles (b) in vials before and after magnetic separation using an external magnetic field, while Figure 1(c) shows the dried fabricated materials.

### 3.2. Characterisation of the Fabricated Materials

The catalytic power of the photocatalysts is affected by the size of the nanoparticles, the distribution of the sizes, and the morphology, since the size of nanoparticles has a strong effect on the energy levels of the photocatalysts [65–67]. Therefore, to obtain a good understanding of the photocatalytic processes, the size of the particles and the morphology should be studied. The fabricated materials were thus characterised using different techniques, namely, TEM analysis and SEM analysis. In addition, compositional analysis was performed using energy-dispersive X-ray analysis (EDAX). For phase identification and the structural analysis of the magnetic nanoparticles, an X-ray diffraction (XRD) instrument was utilised.

#### 3.2.1. TEM Analysis

**Figure 2:**
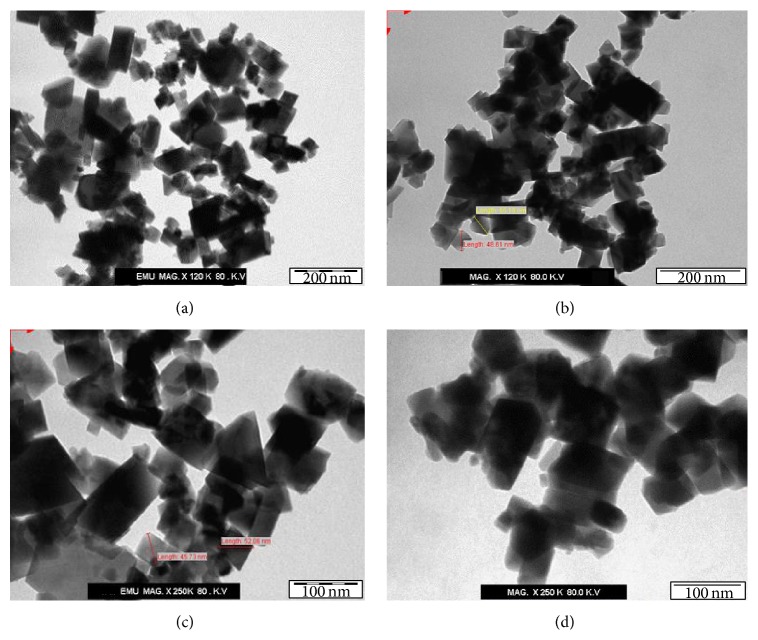
TEM micrographs of naked Fe_3_O_4_ nanoparticles ((a), (c)) and Ag-doped Fe_3_O_4_ nanoparticles ((b), (d)) at different magnifications.

**Figure 3:**
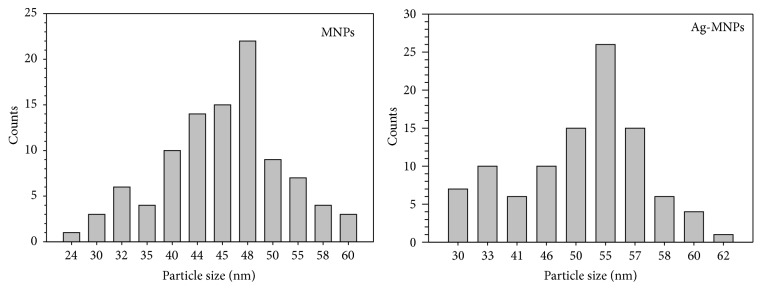
The size distribution histograms of MNPs and Ag-MNPs corresponding to TEM images (a)–(d) shown in Figure 2. The size distribution was obtained by counting 100 nanoparticles for each sample.

A transmission electron microscope is a good tool for extraction of the size and shape data because it yields real images from which measurements can be made [34, 68, 69]; therefore, this was used in order to recognise the morphology and the size of the prepared materials.  Figure 2 shows TEM micrographs of the unsupported Fe_3_O_4_ nanoparticles and the Ag-doped Fe_3_O_4_ nanoparticles using different magnifications. Although the fabricated magnetic nanoparticles were crystalline and tended to have identifiable shapes (rectangles and squares), there were some differences in the comparison. The TEM images showed that the magnetic nanoparticles appeared darker after coating with Ag and they seemed more compact compared with Fe_3_O_4_ nanoparticles, indicating that Ag-doped Fe_3_O_4_ nanoparticles had a higher density than that of Fe_3_O_4_ nanoparticles. The particle size distribution histograms for MNPs and Ag-MNPs have been determined from TEM images (Figure 2), and the corresponding size distribution histograms are shown in Figure 3. The average size of the MNPs was 48 nm within size ranging from 24 to 60 nm. The size distribution of Ag-MNPs ranged from 30 nm to 62 nm, within average size of 55 nm. Commonly, the average diameter of coating MNPs will increase in the range of 0–5 nm compared with the naked MNPs [51]. It was found that the TEM analysis served as an important technique for providing evidence of the formation of the Ag-doped Fe_3_O_4_ nanoparticles.

#### 3.2.2. SEM-EDAX Analysis

**Figure 4:**
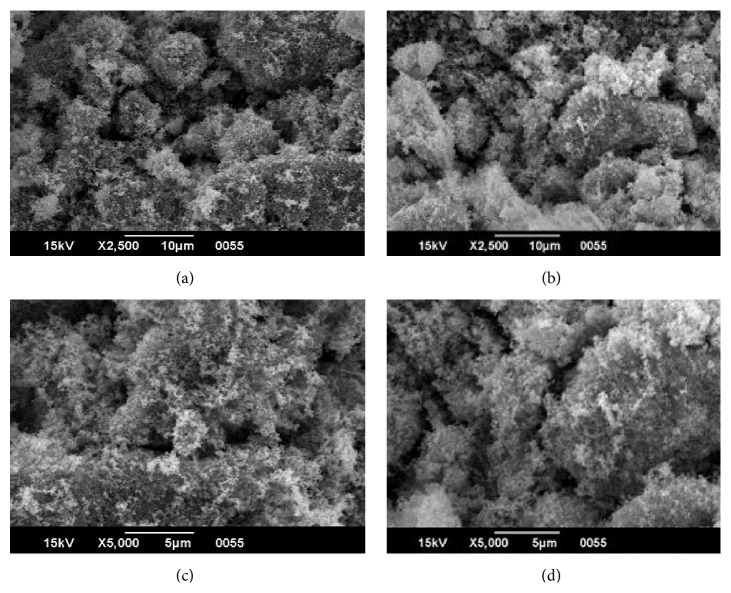
SEM micrographs of Fe_3_O_4_ nanoparticles ((a), (c)) and Ag-doped Fe_3_O_4_ nanoparticles ((b), (d)) at different magnifications.

The morphology and structures of the Fe_3_O_4_ nanoparticles and the Ag-doped Fe_3_O_4_ nanoparticles were also investigated by SEM analysis.  Figure 4 shows the SEM micrographs of the Fe_3_O_4_ nanoparticles and the Ag-doped Fe_3_O_4_ nanoparticles in different magnifications, which suggest that the prepared magnetic nanoparticles have an irregular crystalline form, and furthermore, the surface morphology analysis demonstrates the agglomeration of many ultrafine particles. In addition, no significant morphological differences could be observed between the Fe_3_O_4_ nanoparticles and the Ag-doped Fe_3_O_4_ nanoparticles.

**Figure 5:**
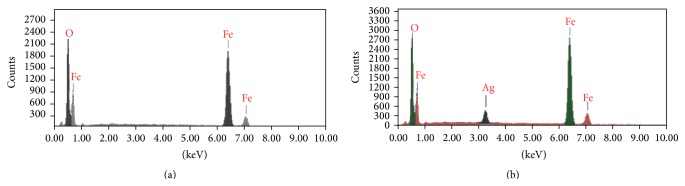
EDAX of (a) Fe_3_O_4_ nanoparticles and (b) Ag-doped Fe_3_O_4_ nanoparticles.

In order to further identify the chemical composition of the fabricated materials, EDAX was utilised to recognise the distribution of Fe, O, and Ag atoms in the prepared materials. The characterisations of the fabricated Fe_3_O_4_ nanoparticles and Ag-doped Fe_3_O_4_ nanoparticles using EDAX are shown in Figure 5. The EDAX spectra showed strong peaks of Fe and O. It was observed that there was a new peak in the Ag-doped Fe_3_O_4_ sample, representing Ag, which confirms the doping of the magnetic nanoparticles with silver.

#### 3.2.3. XRD Analysis

**Figure 6:**
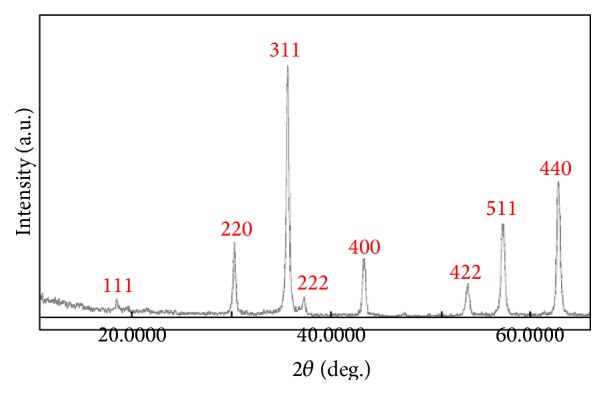
XRD pattern of Fe_3_O_4_ nanoparticles.

The obtained samples were analysed by X-ray diffraction (XRD) using a diffractometer with high-intensity CuK_*α*_ radiation (*λ* = 1.54065 Å) and measured from 10° to 70°, and the results are depicted in Figure 6. For the Fe_3_O_4_ diffraction peaks, seven characteristic peaks at 30°, 35°, 37°, 43°, 53°, 57°, and 62° were seen, corresponding to the (220), (311), (222), (400), (422), (511), and (440) crystal planes of pure Fe_3_O_4_ with a cubic spinel structure of the magnetite [63, 70, 71]. No characteristic peaks of impurities were detected in the XRD pattern, indicating the high purity of fabricated materials. The XRD patterns of Ag-doped Fe_3_O_4_ did not show diffraction peaks due to the doping of Ag, since there was no peak of Ag observed; therefore, it is suggested that the silver dopant was merely placed on the surface of the crystals. The same results have been obtained by [72].

### 3.3. Photodegradation of Eosin Y 

#### 3.3.1. Comparison between the Naked MNPs and the Ag-MNPs Nanoparticles

An organic dye, namely, eosin Y, in aqueous solution was utilised in order to check the performance of the fabricated materials, and their performance was compared with naked magnetic nanoparticles. The photodegradation experiment was performed by adding 50 mg of catalyst to 100 mL of the dye solution (4 × 10^−4^ M). The suspension was magnetically stirred at 600 rpm in the dark for 1 h in order to attain adsorption-desorption equilibrium before the irradiation by UV light (365 nm). The main advantage of using the UV light was to produce electron and hole pair (e^−^/h^+^) with high energy state that migrate to the nanoparticle surface and initiate a wide range of chemical reactions [73].

**Figure 7:**
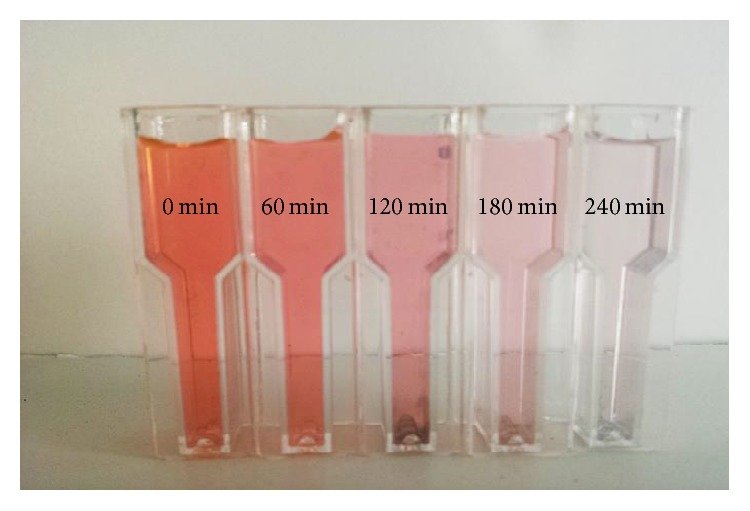
Photographs of eosin Y after treatment with the synthesised Ag-Fe_3_O_4_ powder at different time intervals; the colour of eosin Y solution faded, indicating degradation of the dye.

The suspension was magnetically stirred throughout the experiment. For different irradiation times (0–240 min), at every 60 min interval, an aliquot (2 mL) was taken out. It was observed that the intense orange colour of the initial solution disappears gradually and becomes colourless as the irradiation time increases, indicating the degradation of the dye under UV light irradiation, as can be seen in Figure 7.

**Figure 8:**
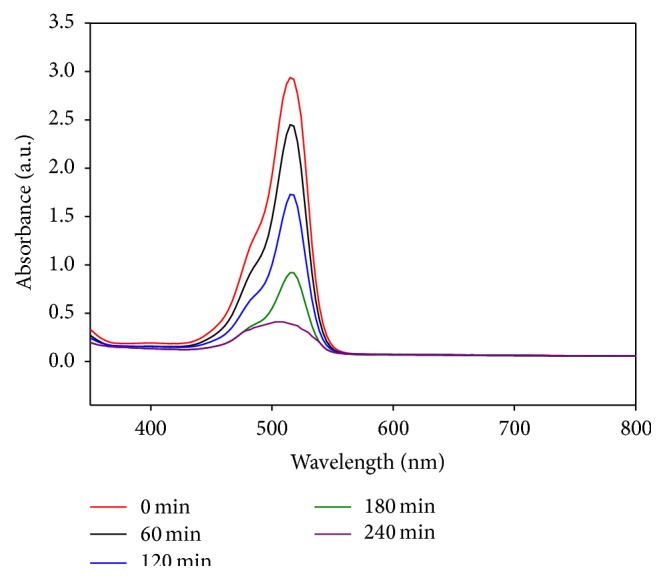
UV-Vis absorption spectra of eosin Y during the course of reaction with the synthesised Ag-MNP powder at different time intervals under ultraviolet light irradiation at room temperature.

The absorption spectra of the dye solution were recorded, as shown in Figure 8, which shows the UV-Vis time-dependent absorption spectrum during the photocatalytic reaction of eosin Y. From the figure, it can be seen that the absorption spectrum maximum of eosin Y steadily decreases as the exposure time of UV light increases from 0 to 240 min, indicating the process of photodecomposition of the dye.

**Figure 9:**
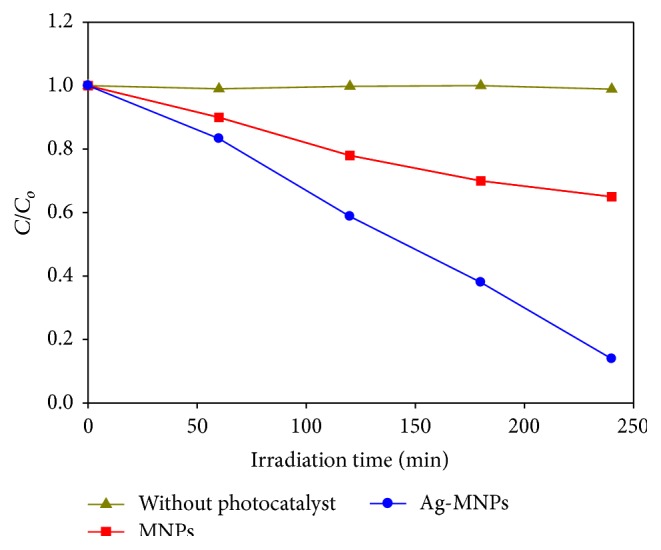
Photodegradation of eosin Y as a function of irradiation time in the absence and presence of MNPs or Ag-MNPs.

The photocatalytic activities of the naked Fe_3_O_4_ nanoparticles and a solution without the photocatalyst, for comparison, were also investigated. It was found that, without adding the photocatalyst, the degradation of eosin Y was very slow at only 5%, while the naked Fe_3_O_4_ nanoparticles showed lower photocatalytic degradation of 40%. The colour removal process of eosin Y using Ag-Fe_3_O_4_ nanoparticles was significantly higher, reaching almost 90.12% decolourisation.  Figure 9 shows the plot of *C*/*C*_*o*_ versus irradiation time. From studying the degradation of eosin Y, it was found that the UV light photocatalytic performance varies in the following order: without catalyst < naked Fe_3_O_4_ nanoparticles < Ag-Fe_3_O_4_ nanoparticles.

**Figure 10:**
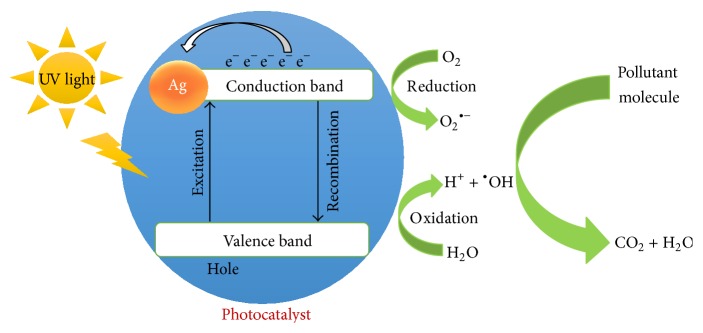
Schematic diagram of the reaction mechanism involved in photocatalytic activity of Ag-MNPs nanoparticles.

Figure 10 represents the mechanism for the role of Ag coating in Fe_3_O_4_ nanoparticles. During the photocatalytic reaction, there will be promotion of an electron from the valence band to the conduction band; thus electron-hole (e^−^/h^+^) pairs will form. The electron in the conduction band is removed by reaction with oxygen dissolved in water while the hole in the valence band can react with water or OH^−^ that are absorbed on the surface of the silver-doped MNPs in order to give hydroxyl radical (^•^OH), which is utilised as a powerful oxidizing agent in order to convert organic pollutants into CO_2_, H_2_O, and less toxic by-products of low molecular weight [73]. The better photocatalytic activity shown by Ag-Fe_3_O_4_ nanoparticles over naked Fe_3_O_4_ nanoparticles can be explained on the basis of silver being acceptor impurity in doping of Fe_3_O_4_. Ag acts as an electron trap and prevents the electron hole recombination that is an important factor in determining the photocatalytic activity [72, 74].

#### 3.3.2. Kinetic Analysis

**Figure 11:**
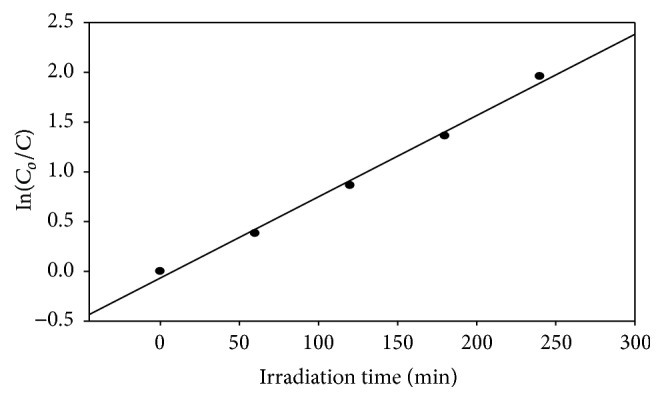
Plot of In(*C*_*o*_/*C*) versus irradiation time.

The rate of reaction in the photodegradation experiment was calculated using pseudo-first-order kinetics: In(*C*_*o*_/*C*) = *kt*, where *C*_*o*_ and *C* are the absorption measured at different illumination times, *k* is the reaction rate, and *t* is the reaction time during the decomposition of eosin Y [75–79]. The reaction rate (*k*) for the photodecomposition of eosin Y was calculated by drawing a graph between In(*C*_*o*_/*C*) and *t*.  Figure 11 presents the relationship between In(*C*_*o*_/*C*) and *t* as a function of irradiation time in the presence of Ag-Fe_3_O_4_ nanoparticles. The figure shows that the photocatalytic degradation follows perfectly the pseudo-first-order kinetics, and the fairness of the fit is indicated by the linear regression value, *R* ^2^ = 0.9938.

#### 3.3.3. Reuse of the Photocatalyst

**Figure 12:**
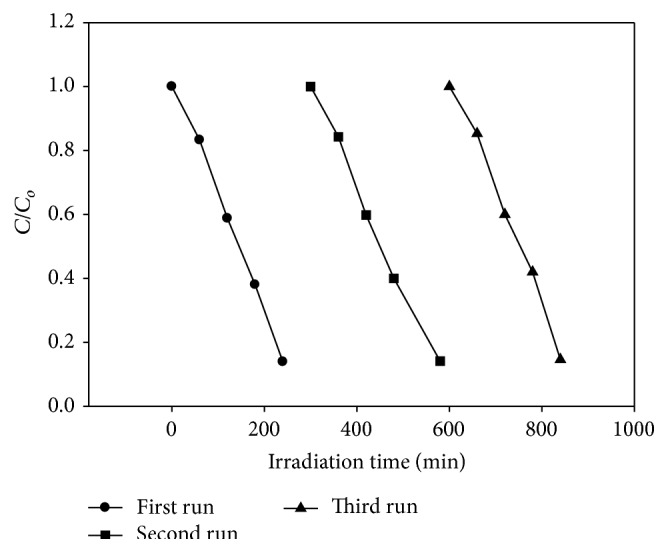
Recycled photocatalytic degradation of eosin Y.

The reuse and the stability of Ag-Fe_3_O_4_ nanoparticles were studied in order to see the cost-effectiveness of the method. This was performed in a very simple way. After the degradation of eosin Y, the Ag-Fe_3_O_4_ nanoparticles were separated easily from the solution using a magnet, while the supernatant was decanted. Then, the magnetic nanoparticles were washed with distilled water and reused for degradation with a fresh lot of eosin Y solution. As can be seen in Figure 12, after using the Ag-Fe_3_O_4_ nanoparticles for three times, the photodegradation of eosin Y did not show any significant loss of activity. These results confirmed that the Ag-Fe_3_O_4_ nanoparticles are not photocorroded during photocatalytic oxidation of the dye.

## 4. Conclusions

Photocatalysis has been ascertained to be a promising method for the removal of harmful dyes from industrial wastewater. In this study, the fabrication of MNPs with coating of Ag was successfully performed. The morphology of the fabricated magnetic nanoparticles before and after the modification with silver was characterised using TEM analysis to conduct the size investigations; SEM analysis was carried out for the surface morphology analysis and EDAX for the compositional analysis. The crystalline structures of the nanoparticles were identified with XRD, which showed the XRD patterns of the Fe_3_O_4_. The experimental results showed that the Ag-MNPs were more effective in enhancing the photocatalytic degradation of eosin Y over naked MNPs. Moreover, the fabricated materials were easily recovered by using an external magnet after the treatment of eosin Y. It is suggested that the UV/Ag-Fe_3_O_4_ system could be used as a useful technique for the removal of harmful dyes from industrial wastewater. Work is currently investigating different azo dyes using the fabricated Ag-MNPs nanoparticles.

## Figures and Tables

**Table 1:** The characteristics of eosin Y dye.

Chemical name	2-(2,4,5,7-Tetrabromo-6-oxido-3-oxo-3*H*-xanthen-9-yl)benzoate

Chemical formula	C_20_H_8_Br_4_O_5_

Chemical structure	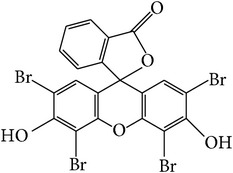

Molecular weight	647.89 g mol^−1^

Type of dye	Anionic dye

*λ*_max⁡_	515 nm
